# Ultrasound mediated accelerated Anti-influenza activity of Aloe vera

**DOI:** 10.1038/s41598-018-35935-x

**Published:** 2018-12-12

**Authors:** Enkhtaivan Gansukh, Judy Gopal, Diby Paul, Manikandan Muthu, Doo-Hwan Kim, Jae-Wook Oh, Sechul Chun

**Affiliations:** 1 Department of Bioresource and Food Science, Konkuk University, Seoul 143-701, Korea Konkuk University, Seoul, 143-701 Korea; 2 Department of Environmental Health Sciences, Konkuk University, Seoul, 143-701 Korea; 3 Pilgram Marpeck School of Science, Tech., Engineering, and Mathematics, Truett McConnell University, Georgia, 30528 United States; 4 Department of Animal Biotechnology, Konkuk University, Seoul, 05029 Korea

## Abstract

*Aloe vera* (AV) is popular and has been commercialized as a beauty product, laxative, herbal medicine, the antimicrobial activity of AV is proven. The antiviral activity of AV however, has not been well documented except for a handful reports. Till date extraction of AV compounds is popularized using organic solvents, since the active components are effectively extracted in methanol. In the current work, we have employed a 5 min ultrasound based extraction for the effective extraction of aloin and aloe-emodin compounds from AV in water. This rapid, one-pot extraction process resulted in enhanced extraction of flavonoids and phenolics and enrichment of the aloin and aloe-emodin moieties in the ulrasonicated water extracts. The extracts were tested for their anti-influenza activity and, the results showed that the ultrasound extraction enabled the water extracts to show excellent anti influenza activity comparable to that seen in the methanolic extracts. Compared to the methanolic extracts which showed high cytotoxicity, the water extracts showed zero cytotoxicity. Spectrophotometric scans of the extracts confirmed the enrichment of the aloin and aloe emodin peaks in the ultrasonicated extracts of AV, suggesting their handiwork behind the anti-influenza activity. The demonstrated technique if appropriately implicated, would lead to promising solutions in the pharmaceutical pursuit against influenza virus.

## Introduction

In recent years, the focus on phytomedicine has increased all over the world and an inclination towards medicinal plants and their metabolites as pharmaceutical reserves has been gaining ground. *Aloe vera* is well known and is in use for centuries now for its health, beauty, medicinal and skin care attributes and is already a portion of a variety of commercial products. *Aloe vera*, belonging to the family Liliaceae, is a perennial herb with 30–60 cm long juicy leaves, and is found growing in temperate climates in many parts of the world. Aloe produces many metabolites in high yields and some have been shown to possess useful biological activities. Many of the medicinal effects of Aloe leaf extracts have been attributed to the polysaccharides found in the inner leaf parenchymatous tissue. This Aloe gel consists of 96% water while the remaining 4% contains substances including polysaccharides Vitamins A, B, C, E, calcium, amino acids and enzymes^1^. The bio active compounds of Aloe are used as an astringent and are well known for their haemostatic, antidiabetic, antiulcer, anti-septic, antibacterial, anti inflammatory, antioxidant and anti cancerous properties.

Till date, more than 200 bioactive chemicals have been found in *Aloe vera* (AV) gel^2^ and their biological activities are more likely to be due a synergistic action of the compounds rather than a single compound^3^. Therefore, suitable extraction of the bioactive compounds is vital in order to harness all the active ingredients for their physiological and pharmaceutical properties.

Aloe gel has been found effective against both gram positive and gram negative bacteria and certain fungi and viruses^4–6^. Inhibitory effects of *A*. *vera* against human cyto megalovirus, herpes simplex virus type 2 (HSV-2), and poliovirus have been reported^7^. Influenza is an acute respiratory infection caused by influenza viruses, which has huge global impacts. This virus has the potency to cause severe pandemic and economic loss^8^. Its genome is highly variable having high rate of mutation which make it resistant to many drugs. Currently, synthetic antiviral drugs and methods (nucleic acid protein inhibitors, neuraminidase inhibitors, ion channel blockers and siRNA technique) have limited use due to the emergence of resistant strains, the high cost and the harmful side effects^9^. Herbal drugs are of low cost and low toxicity and, usually have multi-target effects, which not only act as antiviral agents but also stimulate immunity system to resist the virus^10^. The classically defined antiviral mechanisms for medicinal plants include inhibiting virus replication, blocking virus attachment, inactivating the virus and prevention from virus infection^11^. Interest in employing these agents has been enhanced by investigators and clients due to preference for natural medicines and concerns about the toxic effects of synthetic materials.

Ultrasound is characterised by high (18 kHz–1 MHz) frequency, small (less than about 50 mm) displacements, moderate (a few m s^−1^) velocities, steep transverse velocity gradients (upto 4,000 s^−1^) and very high (up to about 80,000 g) acceleration. Ultrasonication produces cavitation when acoustic power inputs are sufficiently high, allowing for microbubbles at nucleation sites. The bubbles grow during the rarefying phase of the sound wave and then collapse during the compression phase. On collapse, a violent shock wave passes through the medium. The entire process of gas bubble nucleation, growth and collapse due to the action of intense sound waves is called cavitation. When it comes to extraction, the use of ultrasound or sonication for extraction via breaking cell membranes has the advantage of reducing considerably the extraction time and increasing the extract yield^12^. The application of ultrasound disrupts the cell wall structure and accelerates diffusion through membranes; thus, the cell lyses and hence facilitates the release of cell contents^13^. Further, Toma *et al*.^14^, have evidenced that enhanced hydration of vegetative tissues during sonication occurs simultaneously with vegetal tissue fragmentation and leads to enhanced extraction during ultrasonication. Thus, ultrasound technology is well established for its promising outputs when it comes to application towards extraction.

The current work focuses on using an ultrasound based methodology for tapping the rich reservoirs of bioactive compounds from AV extracts. The ultrasonic extraction frequency has been optimized for ideal results. Ultrasound extraction created nano-packets of active metabolites that showed enhanced solubility in water. The effective extraction of aloin and emodin components that were previously reported to be extracted best in organic solvents in water using the ultrasound technology is demonstrated for the first time. The anti influenza ability of the extracts was tested and its candidature as a potent anti influenza drug has been established in the following work.

## Materials and Methods

### Preparation of Aloe vera extracts

Fresh specimen of *Aloe vera* leaves were collected from matureplants. *Aloe vera* gel was collected from this plant by removing leaf rinds, after surface sterilization and washing with fresh water for 5 min and then rinsing with sterile distilled water. The rind was cut and removed and the colorless mucilaginous paranchymatous tissue (Aloe vera gel) was scraped out using a sterile scalpel and collected in a sterile container. 2 g of the gel was suspended in 10 ml of sterile water/ethanol/methanol. The samples were vortexed briefly to break the tissue masses. One set of the Aloe in water, ethanol and methanol were maintained as such as Controls (not sonicated) and coded AV-WNS, AV-EtOHNS and AV-MtOHNS. The remaining samples were sonicated using a Bandelin GM 2200, 200 W/20 kHz probe sonicator, to 25%, 50% and 100% sonication frequencies for 5 min. These samples were coded AV-W25, AV-50 and AV-W100; AV-EtOH25, AV- EtOH50 and AV- EtOH100 and AV-MeOH25, AV-MeOH50 and AV-MeOH100 based on their extractant and sonication frequency. These sample codes will be followed hereon.

### Biochemical characterization of the extracts

#### UV- Visible Spectrophotometer

The extracts were characterized using a Nanodrop ND-1000 v 3.3.1 spectrophotometer, (Nanodrop Technologies, Inc., Wilmington, USA). The absorbance was scanned from 220–700 nm.

#### Total phenolics

The phenolic compounds were estimated following standard procedures described in Shang *et al*.^15^. Briefly, 50 µL of the respective extracts were mixed with Folin and Ciocalteu’s phenol reagent. After 7 min, 600 µL of 20% sodium carbonate solution and 1150 µL of distilled water were added to the mixture and thoroughly mixed and the reaction kept in dark for 60 min and absorbance measured at 765 nm. Gallic acid was used to obtain the standard curve (0.0325–0.5 mg mL^−1^, y = 2.4005x + 0.0107, R^2^ = 0.9941) and the results were expressed as mg of gallic acid equivalents per milliliter (mg GAE per mL).

#### Total flavonoid

Flavonoid contents were measured using the colorimetric method according to Chang *et al*.^16^, with slightly modifications. Briefly, the extracts (0.5 mL) were mixed with 0.1 mL of 10% aluminum chloride solution, 0.1 mL of 0.1 M potassium acetate and 2.8 mL of distilled water and allowed to stand for 30 min at room temperature. The absorbance was measured at 415 nm. Rutin was used as standard (15.15–500 µg mL^−1^, y = 0.0033x − 0.0017, R^2^ = 1), and the results were expressed as µg rutin equivalents per mL (µg RE per mL).

#### Antioxidant activity

The antioxidant activities of the extracts was determined using 1,1-diphenyl-2picrylhydrazyl (DPPH) assay. The DPPH radical scavenging activity test was determined following Cheung *et al*.^17^ and Shang *et al*.^15^. The reduction of DPPH radicals was estimated by measuring the absorption at 517 nm. The percentage of DPPH scavenging activity (AA%), was calculated using the equation:AA% = 100 [(A_sample_ − A_blank_)/A_control_], where A_control_ is the initial absorbance of the methanolic DPPH solution, and A_sample_ is the reaction mixture at 515 nm (DPPH + sample).

### Evaluating the Anti-influenza ability of Aloe extracts

#### Antiviral activity and cytotoxic detection assay

Antiviral activity and cytotoxicity were determined by SRB assay with the cytopathic effect (CPE) reduction method as previously reported^18^.The Madin-Darby Canine Kidney (MDCK) cells (1.5 × 10^4^) were seeded and incubated for a period of 24 h in a 96-well plate. The cells were washed twice with phosphate buffered saline (PBS) and the influenza AP/R/8 virus solution (virus stock solution diluted (4 × 10^3^) with DMEM contained trypsin–EDTA) was used for influenza infection. The prepared virus solution (90 μL/TCID_50_ explained by Gansukh *et al*.)^18^ and aloe extracts (10 μL) of different concentrations (total concentration: 1, 2, 4 and 8 mg/mL) were placed onto the 96 well plates and each sample triplicated into three independent wells. Plates are maintained for 48 h in a humidified CO_2_ incubator. After 48 h of incubation, the culture medium was removed and washed twice with PBS solution before fixing the cells. The cells were fixed using 70% cold acetone for 1 h at −4 °C and then oven dried at 60 °C. Fixed and dried plates were used for the Sulforhodamine-B (SRB) assay to capture the cellular morphology and collect spectral data. Cytotoxicity of the different aloe extracts was tested on normal MDCK cells. The cells were cultured in 96 well plates (1.5 × 10^4^) and maintained for 24 h in a cell culture incubator. And then the cells were washed with PBS and the medium renewed (excluding virus solution). Washed cells were incubated along with aloe extracts in different concentrations (total concentration: 1, 2, 4 and 8 mg/mL). Fixations and SRB determinations were done similar to that of the virus evaluation assay.

#### SRB assay

**Figure 1:**
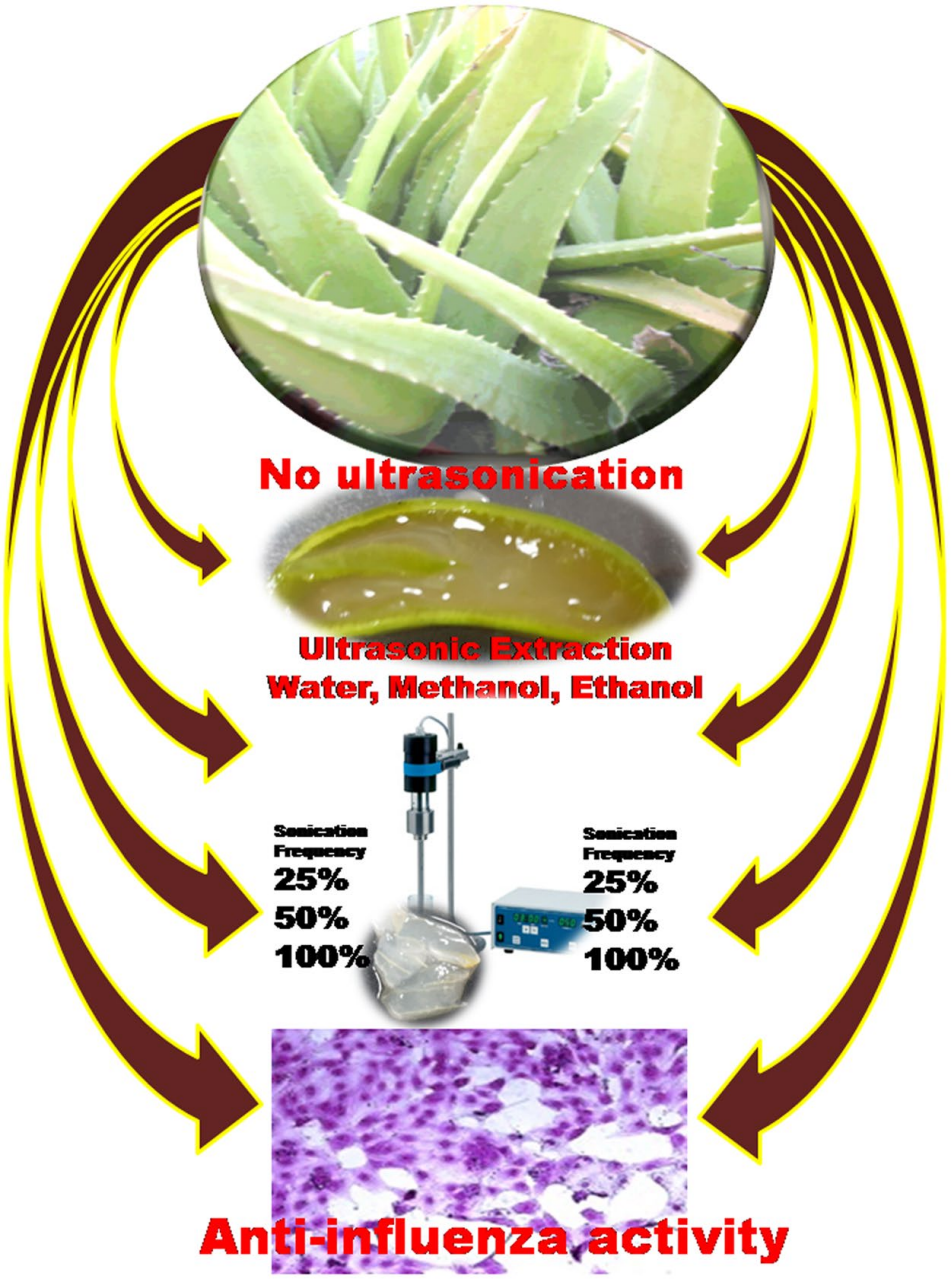
Schematic representation of the flow of work.

The SRB assay was carried out by adding 100 μL of SRB (0.4 mg/100 mL) solution to the tested 96 wells and washed three times with 1% acetic acid^18^. The 96 well plates were dried in the hot air oven and the cell morphology was imaged using abright field microscope at 40× magnification. The images were contrasted to determine the antiviral activity. SRB bound to the cells were dissolved in100 µL Tris base solution (10 mM). The concentration of SRB in each of the wells were captured by UV-spectrophotometer at 510 nm to determine the 50% virus inhibition concentration (IC50), 50%cytotoxic concentration (CC50) and therapeutic index (TI) values of the 12 aloe extracts. Figure 1 represents the schematic experimental work flow of this study.

## Results and Discussion

### Ultrasonication on AV extracts

**Figure 2:**
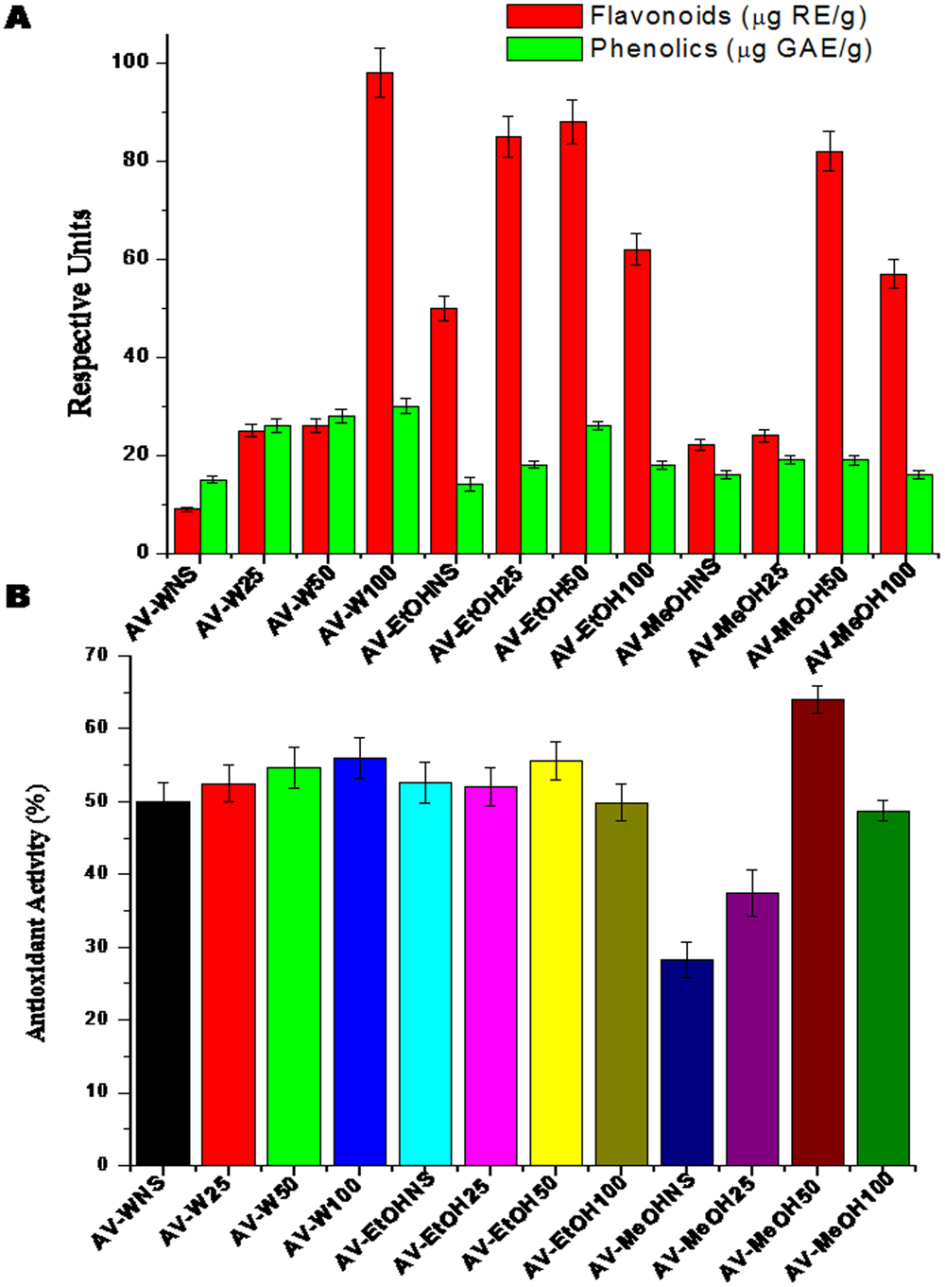
Phytochemical analysis and antioxidant ability of various Aloe extractions. (**A**) Total phenolic and total flavonoid content of Aloe extracts extracted by different methods. (**B**) The DPPH radical scavenging activity of Aloe extractions. Each value was obtained from an average of three independent experiment standard deviation.

All the AV extracts including the non-sonicated (AV-WNS, AV-EtOHNS and AV-MeOHNS) samples and their sonicated counterparts AV-W25, AV-50, AV-W100; AV-EtOH25, AV- EtOH50, AV- EtOH100 and AV-MeOH25, AV-MeOH50, AV-MeOH100 were characterized for their bioactivity using flavonoid and phenolic assays and their corresponding antioxidant activity compared using the DPPH assay. Fig. 2A displays the results of the flavonoid and total phenolic assays. As observed from the graph, it can be observed that compared to the non sonicated, AV-WNS, AV-EtOHNS and AV-MeOHNS all the ultrasonicated extracts (AV-W25, AV-50, AV-W100, AV-EtOH25, AV- EtOH50, AV- EtOH100, AV-MeOH25, AV-MeOH50, AV-MeOH100) showed higher flavonoid and phenolic contents. Ultrasound extraction was found to significantly enhance the bioactive flavonoid and total phenolics in the AV extracts. Several reviews^19–23^ on the application of ultrasonication for extraction of bioactive compounds from herbs and various plant sources, have confirmed the applicability of this technique. Generally, high intensity (>10 W) ultrasound is known to lyse cells in suspensions^23–29^ and increase the permeability of their membranes, thus spilling their contents. It is also reported that ultrasonic waves generate microcavitations in the liquid surrounding the plant material. This leads to mechanical disruption of the cell wall releasing the contents and resulting in local heating of the liquid, increasing diffusion. The kinetic energy and ultrasound waves following the collapse of cavitation bubbles at the solid-liquid interfaces improve mass transfer across cell barriers^30^. In line with these reputations, it is not doubt that ultrasound is able to rupture cell walls and release cellular contents, leading to effective and rapid extraction of bioactive compounds. The efficacy of sonication based extraction is reflected by the 2–3 fold increase in the extraction of the bioactive flavonoids and phenolics. Further, it is also known that conventional extraction methods have been associated with high solvent requirements of toxic organic solvents, usually required long extraction time which increased the risk of degradation of thermo-labile constituents thus resulting in lower extraction yields^31–33^. Ultrasonics has ushered a progress in this area too, where rapid and enhanced water based extraction is achievable^34^. Compared to conventional extraction, this improves the extraction process, decreasing both extraction time and temperature while increasing the rate of extractionof AV^35^. Additionally, from our results we could see that water based extraction yielded equivalent results to that obtained from methanolic or ethanolic extracts. This is a further breakthrough, since current claims hold their ground that active components in AV are better extracted in methanol^32^. The enhanced flavonoids and phenolics obtained when employing ultrasound extraction, dictate enhanced biological activity. Phenolics are major bioactive compounds known for their health benefits and multiple biological effects, including antioxidant activity^36^. Flavonoids belong to a large group of polyphenolic compounds having a benzo-γ-pyrone structure and are ubiquitously present in plants. It is well established that secondary metabolites of phenolic nature including flavonoids are indulging in a variety of pharmacological activities^37,38^. It is also interesting to observe that of the three sonication frequencies tested, namely 25%, 50% and 100%, it was observed that a steep increase in flavonoids and phenolics was observed from 0 to 25% to 50% but between 50% and 100%, no significant increase in the extraction of these bioactive compounds was evident when EtOH and MeOH were used. But in case of water at 100% frequency (AV-W100), highest bioactive compound contents were obtained. In few cases, as in methanolic and ethanolic extracts AV-MeOH100 and EtOH100 even showed marginal decrease compared to MeOH50 and ETOH50. Thus, the optimum sonication frequency for maximum extraction of flavonoids and total phenolic compounds from AV has been optimized as 50% sonication frequency when using solvents and 100% when using water.The overall order of the bioactive compound extraction efficiency with respect to the solvent system was AV-W > AV-MeOH > AV-EtOH.

The antioxidant activity of the extracts following sonication was also assessed using DPPH assay, similar to the results from the flavonoid and total phenolic assays, in this case too it was observed that sonication did not show wide difference in the antioxidant activity, yet compared to the unsonicated samples the ultrasonicated samples showed higher antioxidant activity (Fig. 2B). Moreover, it was observed (Fig. 2B) that AV-MeOH50 exhibited the highest antioxidant activity followed by AV-W100. Earlier authors have reported the relationships between phenolic content and antioxidant activity; while some authors found a high correlation between the phenolic content and the antioxidant activity^39–43^ and others found no such distinct relationship^44,45^. In our study we found a somewhat similar relationship between the phenolic contents and antioxidant activity, with few exceptions. Summarizing these inferences, we can confirm that ultrasonic extraction increased the extraction of bioactive compounds (that positively influences the biological properties of the extracts) from AV.

### Anti-influenza activity of AV extracts

The ultrasonicated and control (non sonicated) extracts were tested against influenza virus. Table 1 presents the results of the antiviral studies. As observed from the table, in case of AV-water extractions, sonication had higher antiviral activitycompared to the unsonicated extracts. IC50 of AV-WNS was 77.16 mg/mL, whilethe IC50 for AV-W25, AV-W50 and AV-W100 was 13.01 mg/mL, 9.69 mg/mL and 4.94 mg/mL, respectively. Lower the IC50 better the antiviral activity of the extract. Thus, the highest anti influenza activity within the water extracts was observed in AV-W100 compared to the rest and it was interesting to observe that at all the concentrations tested (1–8 mg/mL) no cytotoxicity was evident. With respect to EtOH extraction, ultrasonication led to enhanced anti-influenza activity. However, the activity was significantly lesser than that compared to what was obtained from the AV-W50 and AV-W100 extracts. Moreover, cytotoxicity as represented by the CC50 values was pronounced in case of EtOH extracts, while in this aspect AV-W extracts has a clear edge (nil cytotoxicity). In case of MeOH extractions, sonication extracts of AV showed significantly higher anti influenza activity. Significant increase was observed in the order AV-MeOHNS < AV-MeOH25 < AV-MeOH50 < AV-MeOH100. Not much difference was observed in terms of antiviral activity between AV-MeOH50 and AV-MeOH100. Additionally, as observed from the CC50 values, it was observed that cytotoxicity significantly increased in AV-MeOH100. CC50 values of AV-MeOH50 also indicated cytotoxicity, thus although AV-MeOH50 showed significantly higher anti influenza activity compared to the AV-W extracts, yet in terms of zero cytotoxicity, AV-W50 and AV-W100 hold a higher ground compared to AV-MeOH50.

**Table 1:** Consolidation of the anti-influenza tests using AV extracts.

*Test Samples*	*IC*_*50* (± *SD of mean*)_	CC_50_	TI
AV-WNS	77.16 ± 6.83	NA	NA
AV-W25	13.01 ± 2.66	NA	NA
AV-W50	9.69 ± 0.53	NA	NA
AV-W100	4.94 ± 0.57	NA	NA
AV-EtOHNS	13.34 ± 0.84	4.98 ± 0.55	0.4
AV-EtOH25	5.62 ± 0.08	3.44 ± 0.71	1.1
AV-EtOH50	3.24 ± 0.47	3.44 ± 0.32	0.6
AV-EtOH100	3.60 ± 0.54	3.55 ± 0.09	1.0
AV-MeOHNS	10.64 ± 0.04	14.32 ± 0.34	1.3
AV-MeOH25	7.64 ± 0.09	13.76 ± 0.21	1.8
AV-MeOH50	4.89 ± 0.55	13.08 ± 0.33	2.7
AV-MeOH100	4.63 ± 0.09	5.76 ± 0.52	1.2

NA – since water has no toxicity effect, not applicable.

Novel influenza A H7N9 virus and highly pathogenic H5N1 virus, pose challenges to public health and necessitate the pursuit for new anti-influenza compounds. Anthraquinone derivatives like aloe-emodin, emodin and chrysophanol, reportedly exhibit antiviral activity. Previous findings^46^ have recorded the inhibitory effect of 0.2–5% Aloe vera gel (extracted in 2% dimethyl sulfoxide (DMSO)) on herpes simplex virus in Vero cell line. AV as an herbal medicine has been reported to exhibit inhibitory effects against some kind of viruses such as human cytomegalovirus, herpes simplex virus type 2 (HSV-2), and poliovirus^7,47^.

**Figure 3:**
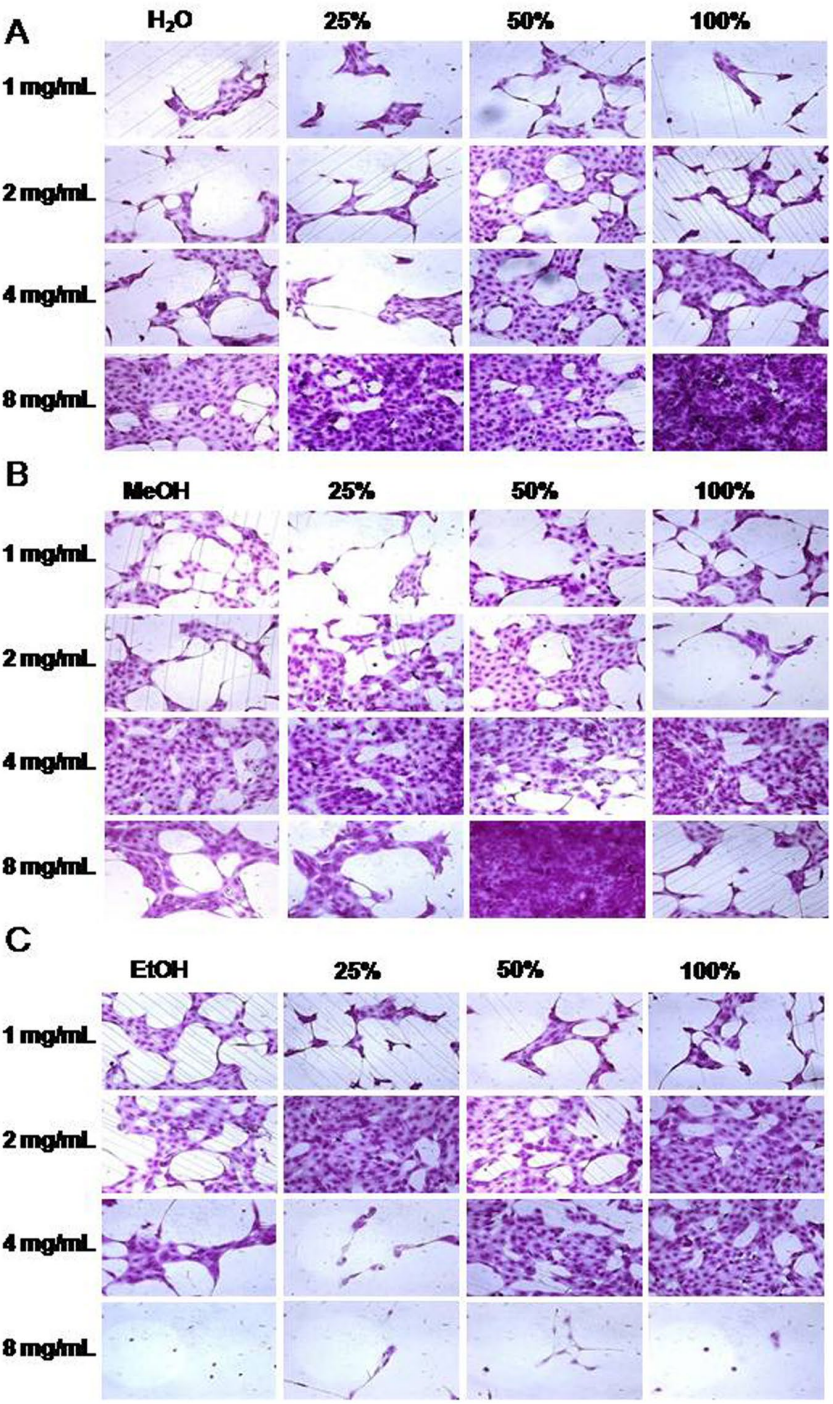
Micrographs showing results of SRB antiviral assay. Influenza A/PR/34/8 (TCID_50_) virus infected MDCK cells (1.5 × 10^4^) were treated with various aloe extracts at various concentrations. After incubating for two days at 37 °C in 5% of CO_2_, infected cell morphology was captured by light microscopy. (**A**) Water extract, (**B**) MeOH extract and (**C**) EtOH extract.

**Figure 4:**
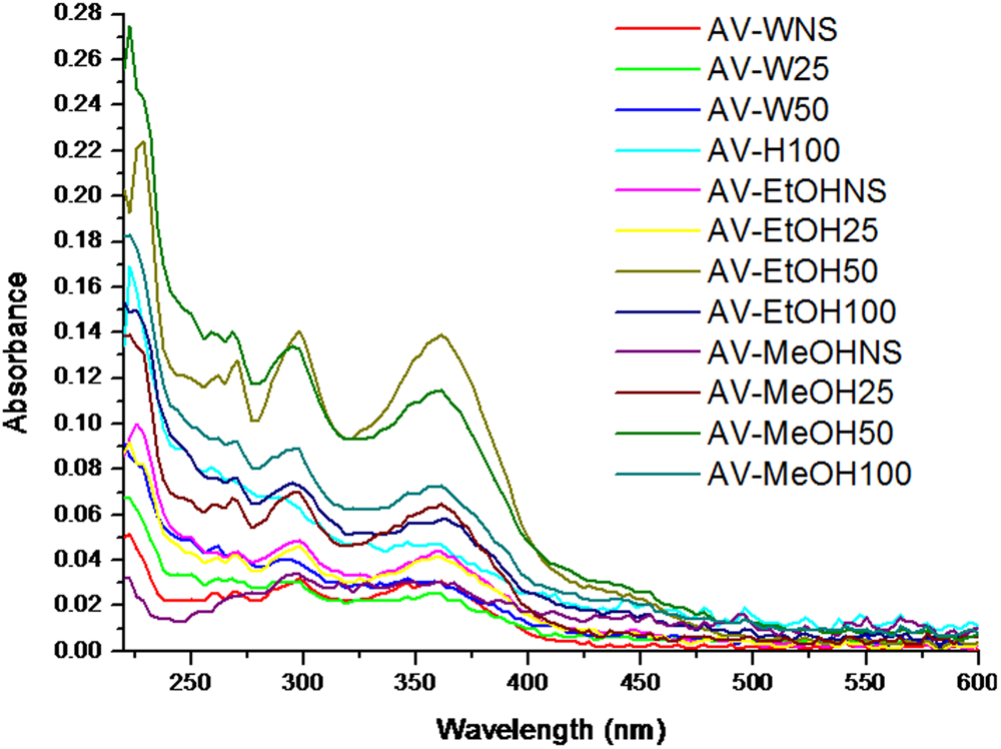
UV-Vis spectra of the various extracts, scanned from 220 to 700 nm, showing UV λ_max_ (nm) absorbance for Aloin peaks at 298, 354 nm; Emodin peaks at 225, 260 and Aloe Emodin peaks at 225, 265.

Although AV is well known for its antimicrobial activity, not much work has been undertaken with respect to antiviral activity and still lesser (just one) with respect to influenza virus. Our results (Fig. 3), strongly indicate that AV possesses highly significant anti influenza activity. Ultrasound extraction of bioactive compounds from AV is believed to have enable this unprecedented activity against this evasive virus. AV contains 99% water and rest polysaccharides and active compounds^48,49^. Aloin is an anthraquinone glycoside. It has molecular weight 418, molecular formula C21H22O9. Its IUPAC name is 8-Dihydroxy-10-(ȕ-D-glucopyranosyl)-3- hydroxymethyl) -9(10 H)- anthracenone^48^. In order to characterize the 12 different extracts obtained prior to and after sonication, we scanned each of these extracts using UV-Vis spectrophotometer from 220 nm to 700 nm. Figure 4 shows the graph revealing the UV-Vis spectra obtained. As observed from the graph, distinct increase in specific peaks at 270 nm, 290 nm and 350–380 nm were observed. Ray *et al*.^50^ have reported that absorption peaks at 255 nm, 270 nm, 290 nm and 350 nm correspond to Aloin. Moreover, it is said that the absorption band between 320 and 380 nm indicates the presence of phenolic compounds^51^. Our results strongly correlate with the above findings and also as shown in the graph, ultrasonication lead to manifold enhancement of Aloin peaks. Especially AV-MeOH50, AV-MeOH100, AV EtOH50 and a lesser extent AV-W100 too showed high absorbance values for Aloin peaks. Aloin is one of the most important member of the anthraquinones present in *Aloe vera* gel, with its anti-oxidative activities of aloin well documented by Tian and Hua^52^.

Aloe emodin (1, 3, 8-trihydroxyanthraquinone) is another anthraquinone and belongs to the variety of emodin present in the aloe latex (an exudate from the aloe plant). It has a marked anti-viral effect *in vitro* against both herpes simplex virus (HSV) type 1 and 2^47^. Of three anthraquinone derivatives, aloe-emodin, with lower cytotoxicity is reported to be capable of inhibiting replication of influenza A in MDCK cells. Emodin has already been shown to have inhibitory effect on replication of enveloped viruses. Small molecules in different plants including phenolics and polyphenols were reported to be as active as anti-herpetic agents^53–56^; compounds such as these are also found in Aloe vera gel. In addition, other components such as emodin, chrysophanic acid and hypericin have demonstrated antiviral activities against hepatitis B virus and poliovirus^53,55^. Emodin UV absorbance peaks are reported to fall in the 220 to 230 regimes, Fig. 4 clearly shows a distinct peak in this region, corresponding to Aloe-emodin. The ultrasonicated extracts were successful in enriching the extracts in these biologically active emodin, aloin and phenolic peaks as demonstrated by our UV-Vis spectrophotometry results. As reported in one of our earlier papers^34^, ultrasonication resulted in the break down of compounds, whereby we achieved nano-curcumin following 5 min ultrasonication a 100% frequency. This nanocurcumin showed enhanced solubility in water, compared to its micro counterparts which show poor solubility in water. It is also suspected that in this case too, the enhanced extraction of Aloin and emodin moieties in water is owing to such nano-sizing effects following ultrasonication.

**Figure 5:**
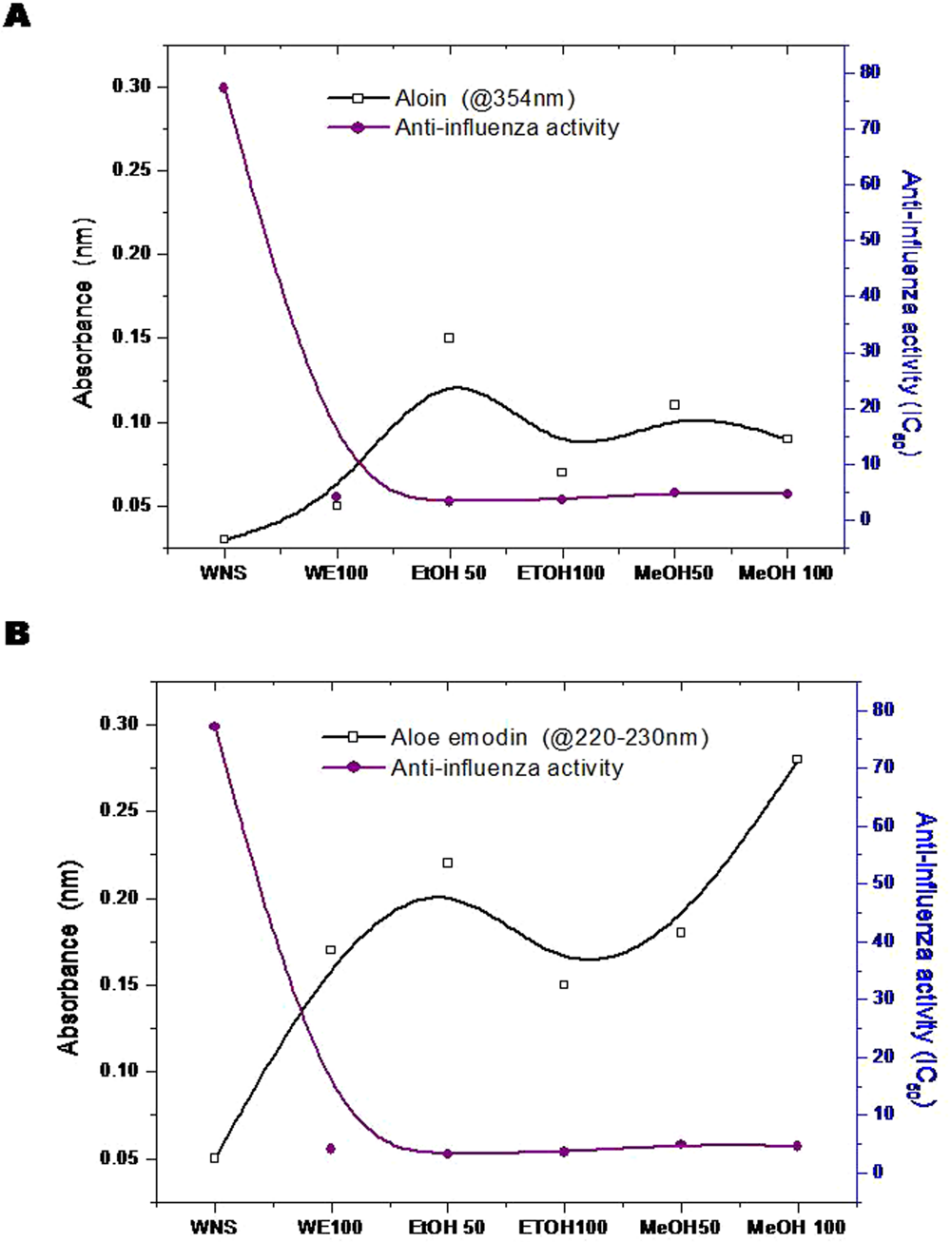
Correlation analysis between Aloin contents (**A**) and Aloe emodin contents (**B**) obtained in various extracts versus anti influenza activity. Aloin and Aloe emodin contents were obtained by measuring their absorption using a UV-Visible spectrophotometer.

**Figure 6:**
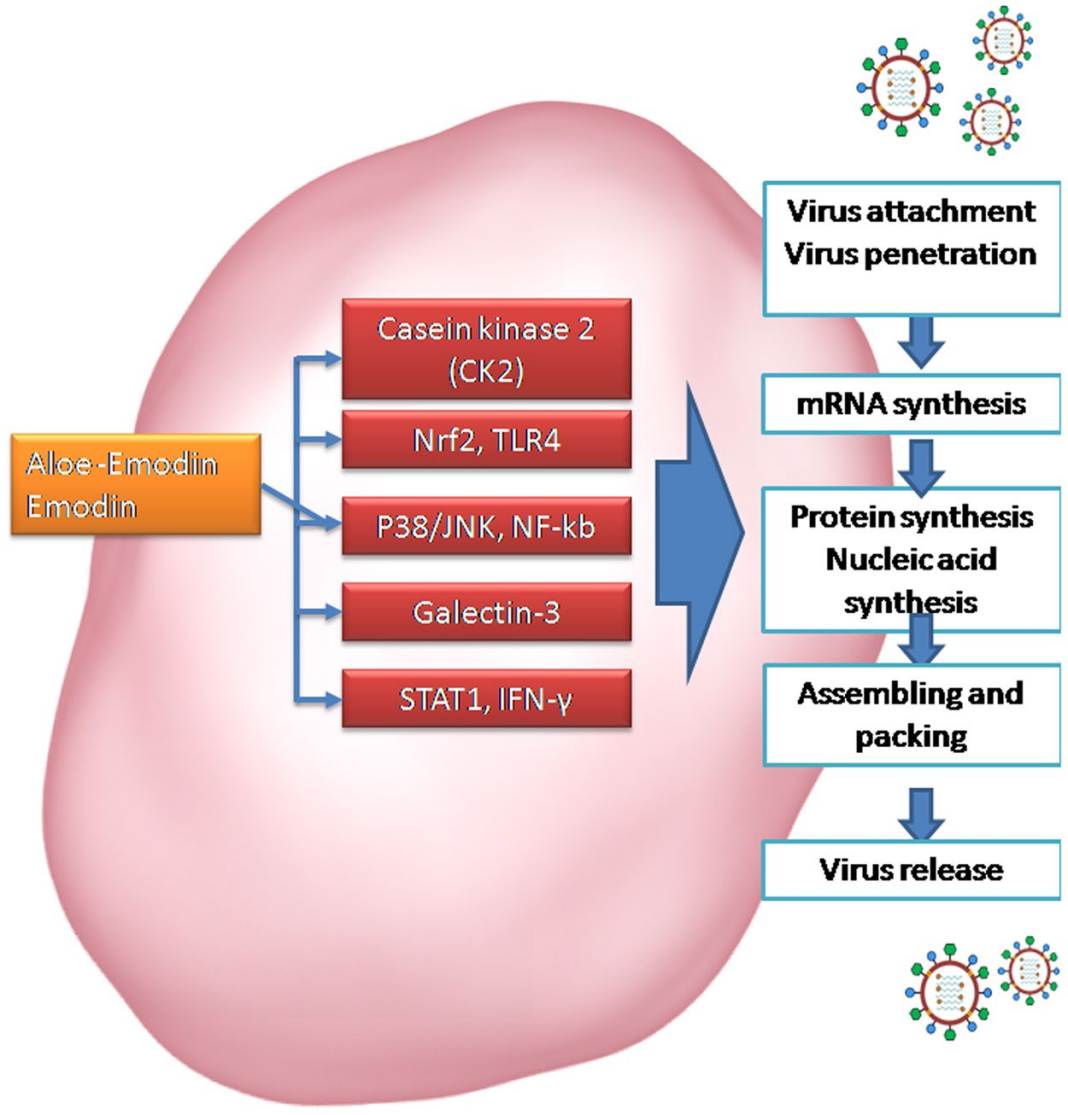
Schematic representation speculating the mechanism behind the aloe emodin based inactivation of the influenza virus. The aloe emodin stimulates cellular mechanisms such as CK2, Nrf2, TLR4, p38/JNK, NF-kb, Galectin-3, STAT1 and INF-γ involved in anti-influenza pathways.

As discussed earlier, these results show a strong correlation to the enhanced presence of aloin and aloe-emodin in the ultrasound extracted AV extracts (AV-W100, AV-MeOH50, AV-MeOH100 and AV EtOH50) and their anti-influenza activities. Especially it was interesting to observe that inspite of AV-W100 having the highest values of aloin, yet its higher aloe-emodin contents, seems to be what helped in its enhanced anti influenza activity. Li *et al*.^53^ have reported that aloe-emodin, with lower cytotoxicity led to successful inhibition of influenza A replication in MDCK cells. Further proteomic and Western blot validation of MDCK cells indicated aloe-emodin to have a hand in up-regulating galectin-3 and thioredoxin and down-regulating nucleoside diphosphate kinase A. Aloe-emodin up-regulated galectin-3 expression; recombinant galectin-3 augmented expression of antiviral genes IFN-β, IFN-γ, PKR and 2′5′,-OAS in infected cells. In turn, galectin-3 inhibited influenza A virus replication. Since galectin-3 exhibited cytokine-like regulatory actions via JAK/STAT pathways, aloe-emodin also restored NS1-inhibited STAT1-mediated antiviral responses in transfected cells^53^. They have nailed aloe-emodin as the key player in the inactivation of influenza viruses. Further, Sydiskis *et al*.^47^ have indicated that aloe emodin directly affected both DNA- and RNA-containing enveloped viruses but had no effect on naked (unenveloped) viruses. They have concluded that emodin acted directly on the envelope of viruses, resulting in the prevention of virus adsorption and subsequent replication. The results showed that aloe emodin inactivated all enveloped viruses tested except adenovirus and rhinovirus. Aloe-emodin was proved for its potent virus inhibitory abilities and high therapeutic indices, in case of HL-CZ cells too, operating via IFN signaling responses against Japanese encephalitis virus and enterovirus 71^55^. The correlation between anti-influenza activity and aloin/aloe emodin was assessed. Figure 5A,B give the results of this correlation. The absorbance at UV λ_max_ (nm) for Aloin (298, 354 nm); Emodin peaks (225, 260 nm) and Aloe Emodin peaks (225, 265 nm)^57^ was plotted as a function of the anti-influenza results obtained in this study. As observed from the graph (Fig. 5A), the contents of aloin in the extracts did not correlate much with the anti influenza activity as much as the contents of Aloe emodin did (Fig. 5B). In those extracts that recorded significant anti influenza activity such as; AV-W100, AV-MeOH50, AV-MeOH100, AV EtOH50 and AV EtOH100, significantly increased Aloe emodin was observed. Also, as observed from the trend, increased Aloe emodin contents were directly proportional to increased anti viral activity. Thus consolidating all these reports and the inferences by Li *et al*., aloe emodin appears to be the key player in the anti- influenza attack reported here and the enhanced extaction of the aloe-emodin compounds resulted in the enhanced anti influenza activity recorded herewith. Figure 6, gives the schematic speculation of the probable mode of inhibition of the emodin moiety on influenza virus. Aloe emodin appears to have interfered with the casein kinase 2 (CK2), Nrf2, TLR4, P38/JNK, NF-kb, Galectin-3, STAT1, INF pathways. One or more of these pathways or a combination of disruption of all these pathways could be instrumental in inhibition of virus attachment, virus penetration, mRNA synthesis, protein synthesis, nucleic acid synthesis, virus assembly and packing of viral units or release of virus, leading to inhibition of influenza virus. The current results pertaining to the mechanism aspects are rather preliminary, more mechanistic studies in this direction would help nail the exact compound involved or whether a synergistic mode of action is operational. A synergistic mode of action has already been suspected by a previous researcher^3^ who worked on AV. However, we suspect a preferentially higher aloe-emodin handiwork behind the anti-influenza activity.

## Conclusion

We have demonstrated the ultrasound mediated extraction of anti-influenza components from AV in water, ethanolic and methanolic extracts. Ultrasound extraction extruded the choicest antiviral compounds, aloin and aloe-emodin and led to exceptionally high antiviral activity recorded only in the cytotoxic solvent options such as MeOH and EtOH but also in the non-cytotoxic solvent, water. This work is intended to arouse pharmaceutical interest in this direction for harnessing the rich reservoirs in *Aloe vera* through ultrasound assisted extraction.
